# Peptide-Like Nylon-3 Polymers with Activity against Phylogenetically Diverse, Intrinsically Drug-Resistant Pathogenic Fungi

**DOI:** 10.1128/mSphere.00223-18

**Published:** 2018-05-23

**Authors:** Leslie A. Rank, Naomi M. Walsh, Fang Yun Lim, Samuel H. Gellman, Nancy P. Keller, Christina M. Hull

**Affiliations:** a Department of Chemistry, University of Wisconsin—Madison, Madison, Wisconsin, USA; b Department of Biomolecular Chemistry, School of Medicine and Public Health, University of Wisconsin—Madison, Madison, Wisconsin, USA; c Department of Medical Microbiology and Immunology, School of Medicine and Public Health, University of Wisconsin—Madison, Madison, Wisconsin, USA; Carnegie Mellon University

**Keywords:** antifungal drug development, emerging pathogens, fungal disease, host defense peptide mimics, nylon-3 polymers

## Abstract

Understanding the dimensions of fungal diversity has major implications for the control of diseases in humans, plants, and animals and in the overall health of ecosystems on the planet. One ancient evolutionary strategy organisms use to manage interactions with microbes, including fungi, is to produce host defense peptides (HDPs). HDPs and their synthetic analogs have been subjects of interest as potential therapeutic agents. Due to increases in fungal disease worldwide, there is great interest in developing novel antifungal agents. Here we describe activity of polymeric HDP analogs against fungi from 18 pathogenic genera composed of 41 species and 72 isolates. The synthetic polymers are members of the nylon-3 family (poly-β-amino acid materials). Three different nylon-3 polymers show high efficacy against surprisingly diverse fungi. Across the phylogenetic spectrum (with the exception of *Aspergillus* species), yeasts, dermatophytes, dimorphic fungi, and molds were all sensitive to the effects of these polymers. Even fungi intrinsically resistant to current antifungal drugs, such as the causative agents of mucormycosis (*Rhizopus* spp.) and those with acquired resistance to azole drugs, showed nylon-3 polymer sensitivity. In addition, the emerging pathogens Pseudogymnoascus destructans (cause of white nose syndrome in bats) and Candida auris (cause of nosocomial infections of humans) were also sensitive. The three nylon-3 polymers exhibited relatively low toxicity toward mammalian cells. These findings raise the possibility that nylon-3 polymers could be useful against fungi for which there are only limited and/or no antifungal agents available at present.

**IMPORTANCE** Fungi reside in all ecosystems on earth and impart both positive and negative effects on human, plant, and animal health. Fungal disease is on the rise worldwide, and there is a critical need for more effective and less toxic antifungal agents. Nylon-3 polymers are short, sequence random, poly-β-amino acid materials that can be designed to manifest antimicrobial properties. Here, we describe three nylon-3 polymers with potent activity against the most phylogenetically diverse set of fungi evaluated thus far in a single study. In contrast to traditional peptides, nylon-3 polymers are highly stable to proteolytic degradation and can be produced efficiently in large quantities at low cost. The ability to modify nylon-3 polymer composition easily creates an opportunity to tailor efficacy and toxicity, which makes these materials attractive as potential broad-spectrum antifungal therapeutics.

## Abstract (precis)

Fungi reside in all ecosystems on earth and impart both positive and negative effects on human, plant, and animal health. Fungal disease is on the rise worldwide, and there is a critical need for more effective and less toxic antifungal agents. Nylon-3 polymers are short, sequence random, poly-β-amino acid materials that can be designed to manifest antimicrobial properties. Here, we describe three nylon-3 polymers with potent activity against the most phylogenetically diverse set of fungi evaluated thus far in a single study. In contrast to traditional peptides, nylon-3 polymers are highly stable to proteolytic degradation and can be produced efficiently in large quantities at low cost. The ability to modify nylon-3 polymer composition easily creates an opportunity to tailor efficacy and toxicity, which makes these materials attractive as potential broad-spectrum antifungal therapeutics.

## INTRODUCTION

The pervasiveness of fungi and their impact on globally important processes can be seen in the evolution of the human immune system (1), the chemistry of soil (2), the genomes of plants (3, 4), and even the progression of climate change (5). Fungi interact extensively with plants, animals, bacteria, and other organisms; these relationships range from mutualists to saprotrophs to pathogens. The influence of fungi across of variety of biological processes has its roots in the massive degree of diversity within the kingdom of fungi. Fungi are characterized by varied morphologies, diverse growth strategies, and assorted nutrient acquisition capabilities. Though only roughly 100,000 species of fungi are accepted in the current taxonomy (6), it is estimated that there exist on the order of 1.5 million fungal species and that up to one-quarter of the world’s biomass is of fungal origin (7, 8). Our imperfect understanding of fungal diversity arises from the lack of taxonomically relevant morphological features and pleomorphisms of fungi, the difficulty or impossibility of culturing many fungi isolated from natural environments, and the inability to consistently identify diagnostic sexual structures in fungi grown in culture or observed in the environment. The great ecological success of fungi, either as filamentous or unicellular growth forms, is a testament to their diversity and functional importance in the global ecosystem.

Understanding the dimensions of fungal diversity has major implications for disease control, crop management, and decomposition of both recalcitrant organic and synthetic materials. For environmental, agricultural, and biomedical purposes, we need agents that target fungi broadly. The innate immune response to infection by fungi and other microbes includes production of host defense peptides (HDPs), and these molecules offer clues for development of synthetic antifungal agents. HDPs have diverse sequences. Many of these peptides are relatively short (10 to 50 amino acid residues) and adopt globally amphiphilic conformations, although the nature of these conformations is variable (α-helix versus β-sheet versus irregular) (9). HDP amphiphilicity is hypothesized to be critical for disruption of microbial membranes, which results in either microbial killing or inhibition of growth (other antimicrobial mechanisms of action have been proposed as well) (10, 11).

Since their discovery in the 1980s, HDPs and their synthetic analogs have been subjects of interest as potential therapeutic agents. The high cost of production and inherent toxicity to the host manifested by HDPs, however, have discouraged clinical development of these peptides thus far (9). The production challenge has encouraged evaluation of random copolymers with hydrophilic and lipophilic subunits for antimicrobial activity (12–18). Random copolymers can be synthesized in a more economical manner than can sequence-specific peptides.

Nylon-3 polymers have been studied as antibacterial agents since 2007, and more recently, examples with both antibacterial and antifungal activities have been reported (19–23). Nylon-3 polymers are synthesized via the anionic ring-opening polymerization of β-lactams. Use of racemic β-lactams leads to heterochiral polymer chains. The protein-like polyamide backbone of nylon-3 polymers is believed to confer biocompatibility, while the presence of β-amino acid subunits renders these materials resistant to proteolytic degradation (24).

**FIG 1:**
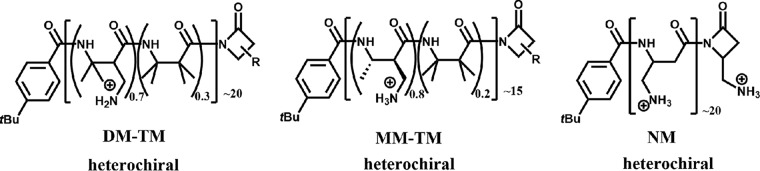
Nylon-3 copolymers employed in this study. All three polymers are heterochiral. *t*Bu, *tert*-butyl.

Three nylon-3 polymers have previously been shown to have activity against pathogenic fungi (20, 22). These polymers, designated MM-TM, DM-TM, and NM (Fig. 1), inhibit the growth of Candida albicans and Cryptococcus neoformans, pathogenic fungi with yeast morphology, as single agents. In contrast, these polymers were able to halt the growth of the pathogenic, filamentous Aspergillus fumigatus only when applied in combination with established azole-based antifungal agents; the polymers were not active as single agents. The activity of MM-TM, DM-TM, and NM against pathogenic yeasts led us to hypothesize that these polymers might be active against a wide range of unicellular yeasts, but not against filamentous fungi. The work described here presents a test of this hypothesis. We have undertaken, to our knowledge, the broadest phylogenetically susceptibility analysis of any antifungal compound to determine the extent of activity of the MM-TM, DM-TM, and NM polymers. We expanded our evaluation to include a diverse array of both filamentous and nonfilamentous species across the fungal kingdom, including members of the *Zygomycetes*, *Ascomycetes*, and *Basidiomycetes* phyla. Surprisingly, most fungi tested, including those naturally resistant to current antifungal drugs, were sensitive to the nylon-3 polymers, which raises the possibility that nylon-3 polymers could be useful against pathogens for which there are only limited and/or no antifungal agents available at present.

## RESULTS

### NM, MM-TM, and DM-TM are active at low concentrations against phylogenetically diverse yeasts.

To evaluate the antifungal activity of MM-TM, DM-TM, and NM against six species of vegetatively growing yeasts across three different genera, we used the CLSI M27-A3 broth microdilution method (Table 1) (25). Polymers were evaluated by measuring MICs (MIC_100_s). For comparison, we also evaluated the commonly used antifungal drug fluconazole (FLC) in terms of MIC_50_ (the MIC to halt 50% of growth per the CLSI M27-A3 standard). All of the yeasts tested were sensitive to all three of the nylon-3 polymers, including those strains resistant to azoles. The polymers were particularly effective against *Cryptococcus* spp. (Cryptococcus neoformans and Cryptococcus amylolentus) of the *Basidiomycota* phylum, exhibiting MIC_100_ values from 2 to 4 µg/ml, which is comparable to or better than concentrations of fluconazole required to halt only 50% of fungal growth. MM-TM, DM-TM, and NM were also active against two genera within the *Ascomycota* phylum: *Candida* and *Saccharomyces*. Saccharomyces cerevisiae was particularly sensitive to all three nylon-3 polymers, with NM being the most active (MIC = 2 µg/ml) and DM-TM the least active (MIC = 8 µg/ml). *Candida* spp. were also susceptible to the polymers, with MIC_100_ values for *Candida albicans* and *Candida krusei* generally ranging from 4 to 16 µg/ml. The polymers were also active against *Candida auris*. Invasive and drug-resistant C. auris infections have been increasingly reported in health care facilities around the world (26, 27). The two strains of C. auris tested both showed sensitivity (MIC_100_s of 4 to 16 µg/ml) to all three polymers, with particular sensitivity to NM. Overall, yeasts were highly susceptible to the three nylon-3 polymers tested for efficacy, with NM being the most active polymer against yeasts.

**TABLE 1:** MIC results for MM-TM, DM-TM, and NM against vegetatively growing yeasts

Species^a^	Isolate	Resistance	MIC_100_ (µg/ml)^b^			FLU MIC_50_ (µg/ml)^c^
			NM	MM-TM	DM-TM	
Saccharomyces cerevisiae	W303		2	4	8	<1

Cryptococcus neoformans	CN1		4	4	4	4
	CN2	FLU	4	4	4	>64
	CN3	FLU	4	4	4	>64

Cryptococcus amylolentus	CBS 6273		4	4	2	16
	CBS 6039		4	4	4	16

Candida albicans	ATCC 90028		4	4	4	1
	SC5314		8	16	16	0.25
	CA3	Azole	8	16	64	>64

Candida krusei	QC^e^		4	16	8	32

Candida auris	B11220		4	8	8	4
	C54039	AMB^d^	8	16	16	16

a Candida lusitaniae was tested previously (23) for MIC activity with MM-TM and shown to have an MIC_100_ of 5 µg/ml.

b MIC_100_, MIC resulting in 100% reduction in growth.

c FLU, fluconazole; MIC_50_, MIC resulting in 50% reduction in growth.

d AMB, amphotericin B.

e QC, quality control.

### The *Aspergillus* genus is generally insensitive to nylon-3 polymers, NM, MM-TM, and DM-TM.

Previous reports show that nylon-3 polymers are largely inactive against the pathogenic fungus Aspergillus fumigatus (22). To determine whether this resistance phenotype was specific to A. fumigatus, we assessed the antifungal activity of MM-TM, DM-TM, and NM against 18 different species within the *Aspergillus* genus. For 6 of the 18 *Aspergillus* species examined, none of the three polymers caused any decrease in hyphal growth relative to a no-treatment control (Table 2, boldface rows, rows 1 to 7). A. fumigatus, A. flavus, and A. terreus are considered to be the most pathogenic species of the *Aspergillus* genus (28). It seems possible that the high pathogenicity of these three species is related to their relatively high resistance to the nylon-3 polymers tested. The remaining 12 species of *Aspergillus* evaluated showed low levels of growth inhibition, with MIC_100_s ranging from 8 to >64 µg/ml, with the DM-TM copolymer demonstrating the most promising antifungal activity among the three nylon-3 polymers. While all 18 species of *Aspergillus* were tested according to CLSI M38-A2 methodology (conidia in liquid culture), four species, selected for their varied response to polymer, were also tested for susceptibility to polymer as hyphal clumps in liquid culture. MIC_100_ values against hyphal fragments were determined as the concentration of agent required to prevent hyphal growth, monitored as an increase in optical density at 600 nm (OD_600_), after incubation at 35°C for 48 h. Three of these species, A. fumigatus, A. terreus, and A. flavus, were highly resistant to polymer as conidia, and their hyphal fragments were also insensitive to polymer. The conidia of the fourth species of *Aspergillus* selected, A. nidulans, were susceptible to polymer, with MIC_100_ values ranging from 8 to 16 µg/ml; however, polymer activity was diminished when incubated with A. nidulans hyphal clumps (MIC_100_ values of 32 to >64 µg/ml). As expected from precedent, the efficacy of itraconazole against *Aspergillus* species as hyphal clumps was also greatly diminished relative to treatment of conidia (MIC_100,conidia_ ~ 1 µg/ml; MIC_100,hyphae_ ~8 to >64 µg/ml) (29). Overall, nylon-3 polymers are ineffective against species within the *Aspergillus* genus, regardless of the life form assayed (conidia or hyphae) for sensitivity.

**TABLE 2:** MIC results for MM-TM, DM-TM, and NM against *Aspergillus* species^a^

Species	Cell type	Isolate	MIC_100_ (µg/ml)^b^			
			NM	MM-TM	DM-TM	ITRA^c^
**Aspergillus fumigatus**	**Conidia**	**AF293**	**>64**	**>64**	**>64**	**<1**
**Aspergillus fumigatus**	**Conidia**	**CEA10**	**>64**	**>64**	**>64**	**<1**
	**Hyphae**		**>64**	**>64**	**>64**	**8–16**
**Aspergillus flavus**	**Conidia**	**NRRL3357**	**>64**	**>64**	**>64**	**<1**
	**Hyphae**		**>64**	**>64**	**>64**	**>64**
**Aspergillus terreus**	**Conidia**	**NCCB IH2626**	**>64**	**>64**	**>64**	**<1**
	**Hyphae**		**>64**	**>64**	**>64**	**>64**
**Aspergillus oryzae**	**Conidia**	**Rib40**	**>64**	**>64**	**>64**	**1**
**Aspergillus parasiticus**	**Conidia**	**Su-1**	**>64**	**>64**	**>64**	**<1**
***Neosartorya fischeri***	**Conidia**	**CBS 544.65**	**>64**	**>64**	**>64**	**>8**
Aspergillus nidulans	Conidia	FGSCA4	16	8	8	<1
	Hyphae		>64	64	32	>64
Aspergillus aculeatus	Conidia	CBS 172.66	>64	>64	8	<1
Aspergillus carbonarius	Conidia	DT0115-B6	64	64	64	<1
Aspergillus wentii	Conidia	DT0136-E9	>64	64	32	<1
Aspergillus sydowii	Conidia	CBS 593.65	>64	64	64	2
Aspergillus foetidus	Conidia	CBS 106.47	>64	64	32	2
Aspergillus zonatus	Conidia	CBS 506.65	16	8	8	<1
Aspergillus niger	Conidia	CBS 113.46	64	32	32	<1
Aspergillus glaucus	Conidia	CBS 516.65	>64	64	32	2
Aspergillus brasiliensis	Conidia	CBS 101740	64	48	48	64
Aspergillus clavatus	Conidia	CBS 513.65	>64	>64	>64	64
Aspergillus versicolor	Conidia	CBS 795.97	16	16	8	1

a Note that the first six species (shown in boldface type) indicate that no reduction in growth was observed after incubation with nylon-3 polymer. The remaining 12 species (shown in lightface type) indicate that some reduction in growth was noted in response to MM-TM, DM-TM, and NM, even when the MIC_100_s were >64 µg/ml.

b MIC_100_, MIC resulting in 100% reduction in growth.

c ITRA, itraconazole.

### NM, MM-TM, and DM-TM are active against phylogenetically diverse filamentous fungi.

On the basis of our observation of low sensitivity of *Aspergillus* spp. toward the polymers as single agents, we hypothesized that nylon-3 polymers were intrinsically less effective against filamentous fungi relative to yeasts. To test this hypothesis, we assessed MM-TM, DM-TM, and NM activity against 10 genera of filamentous fungi, following the CLSI M38-A2 methodology (conidia in liquid culture) or a modified CLSI method (hyphal fragments in liquid culture). Surprisingly, MM-TM, DM-TM, and NM were very active against phylogenetically diverse filamentous fungi, with MIC_100_ values of 4 to 8 µg/ml (Table 3). The polymers were much more effective against medically relevant Rhizopus arrhizus (a causative agent of mucormycosis), the emerging pathogen Paecilomyces variotii (30), and Fusarium oxysporum isolates (which, along with A. flavus, is a serious agent of keratitis [31]) than they were against *Aspergillus* spp. Notably, all three nylon-3 polymers were active against *Scedosporium* spp., both Scedosporium apiospermum and *Scedosporium prolificans*. The latter species is an emerging fungal pathogen of both immunocompetent and immunocompromised individuals that is intrinsically resistant to most antifungal drugs (voriconazole [VOR] MIC_100_ > 16 µg/ml); S. prolificans infections are often fatal (32).

**TABLE 3:** MIC results for MM-TM, DM-TM, and NM against filamentous fungi

Species	Cell type	Isolate	MIC_100_ (µg/ml)^a^				
			NM	MM-TM	DM-TM	POS/VOR/ITRA^b^	FLU^c^
Penicillium expansum	Conidia	d1	>64	>64	64	1 (I)	
	Hyphae		>64	>64	64	>64 (I)	
Talaromyces marneffei	Conidia	FRR2161	64	32	8	0.125 (I)	
Paecilomyces variotii	Conidia	QC^e^	4	8	4	0.125 (V)	
Fusarium oxysporum	Conidia	FO1	4	8	8	4 (V)	
Fusarium oxysporum	Conidia	FO_2_	8	8	8	4 (V)	
Fusarium oxysporum	Conidia	FO3	4	8	4	>16 (V)	
Scedosporium apiospermum	Conidia	SA1	2	8	4	1 (V)	
Scedosporium apiospermum	Conidia	SA2	4	4	4	1 (V)	
Scedosporium prolificans	Conidia	LP1	4	4	4	>16 (V)	
Rhizopus arrhizus	Conidia	RA1	16	8	4	0.5 (P)	
Rhizopus arrhizus	Conidia	RA2	8	8	8	0.5 (P)	
Rhizopus arrhizus	Conidia	RA3	8	8	8	0.5 (P)	
Pseudogymnoascus destructans	Conidia	ATC MYA-4855	4–8	2–4	2–4	0.13 (I)	
Trichophyton rubrum	Hyphae	ATCC 28188	>64	64	16	0.5 (I)	
Trichophyton tonsurans	Hyphae	CBS 112818	16	8	8	0.13 (I)	
Microsporum canis	Hyphae	UW10	16	8	16	<0.13 (I)	
Filobasidiella depauperata^d^	Conidia	CBS 7855	>64	>64	>64	1 (I)	32
	Hyphae		>64	>64	>64		32

a MIC_100_, MIC resulting in 100% reduction in growth.

b The MICs for posaconazole (POS), voriconazole (VOR), or itraconazole (ITRA) are shown followed by the drug abbreviation in parentheses: I, itraconazole; V, voriconazole; P, posaconazole.

c FLU, fluconazole.

d MIC assays conducted in SD medium, rather than RPMI 1640.

e QC, quality control.

For the dermatophytes Trichophyton tonsurans, Trichophyton rubrum, and Microsporum canis, MIC_100_ values were determined as the concentration of the agent required to halt hyphal growth, monitored as an increase in OD_600_, after 4 days of incubation at 29°C in RPMI 1640. Mixed efficacy of the nylon-3 polymers was observed across the three species of dermatophytes tested. The highest levels of antifungal activity were observed against T. tonsurans and M. canis (8 to 16 µg/ml), and the lowest levels were observed against T. rubrum (16 to 64 µg/ml) (Table 3). The MIC_100_ of the dermatophyte Pseudogymnoascus destructans was evaluated after 7 days of incubation of conidia at 12°C in RPMI 1640. The activity against P. destructans, the causative agent of white nose syndrome in bats (33), is encouraging because there are limited options for preventing the spread of this devastating pathogen. The facility with which nylon-3 structure can be varied could provide opportunities for the development of topical agents with high specificity for particular fungi.

Strikingly, nylon-3 polymers were ineffective against Filobasidiella depauperata (MIC_100_ > 64 µg/ml [Table 3]), which is closely related to C. neoformans and grows exclusively as filaments (34). Resistance of F. depauperata to nylon-3 polymers was surprising because of the close phylogenetic relationship between C. neoformans and F. depauperata and the consistent sensitivity of C. neoformans isolates. F. depauperata spores and hyphae were both strongly resistant to polymer in SD medium (synthetically defined, minimal medium) (35); this species of fungi did not grow in the RPMI 1640 growth medium specified by CLSI methods. C. neoformans JEC20x21 spores and yeasts were both sensitive to all three polymers in SD medium (see Tables S1 and S2 in the supplemental material). As this level of inactivity against germinating conidia was reminiscent of the results observed with *Aspergillus* species, we asked whether the nylon-3 polymer MM-TM could act synergistically with an azole to inhibit germination of F. depauperata conidia, as had previously been observed with various A. fumigatus isolates. The copolymer MM-TM exhibited strong synergy (ΣFIC [fractional inhibitory concentration] index value of 0.04) with itraconazole against the azole-sensitive strain CBS 7855, resulting in >20-fold decreases in azole and polymer MIC_100_s when the polymers are given in combination (Table 4). The MM-TM copolymer was previously shown to act synergistically with azole-sensitive strains of A. fumigatus (22). Overall, all three nylon-3 polymers demonstrated strong activity against filamentous fungi spanning the fungal tree of life, with the notable exceptions of F. depauperata and the *Aspergillus* genus.

**TABLE 4:** Synergy checkboard results with azoles and MM-TM against germinating Filobasidiella depauperta (CBS 7855) conidia

Test agent(s)	MIC_100_ (µg/ml)^a^		ΣFIC index^b^	FIC interpretation
	Alone	Combination		
MM-TM	>64^c^	1	0.04	Synergistic
Itraconazole	1	<0.06^d^		

a MIC after 72 h for the compound(s) as a single agent or for the combination of the compounds.

b FIC, fractional inhibitory concentration.

c The high off-scale MIC value, >64 µg/ml, was converted to the next highest concentration, 128 µg/ml, for calculation of the FIC index.

d The low off-scale MIC value, <0.06 µg/ml, was converted to the next lowest concentration, 0.03 µg/ml, for calculation of the FIC index.

10.1128/mSphere.00223-18.3 TABLE S1  MIC results for MM-TM, DM-TM, and NM against Cryptococcus neoformans JEC20x21 yeast in either RPMI or SD medium. Download TABLE S1, PDF file, 0.1 MB. Copyright © 2018 Rank et al. 2018 Rank et al. This content is distributed under the terms of the Creative Commons Attribution 4.0 International license.

10.1128/mSphere.00223-18.4 TABLE S2  Concentrations of MM-TM, DM-TM, and NM required to prevent germination of Cryptococcus neoformans JEC20x21 spores in either RPMI or SD medium. Download TABLE S2, PDF file, 0.1 MB. Copyright © 2018 Rank et al. 2018 Rank et al. This content is distributed under the terms of the Creative Commons Attribution 4.0 International license.

### NM, MM-TM, and DM-TM are active against dimorphic fungi.

Given the sensitivity of yeasts and most filamentous fungi to NM, MM-TM, and DM-TM, we hypothesized that dimorphic fungi would be sensitive to nylon-3 polymers. The CLSI M38-A2 broth macrodilution method was used to test all three polymers against *Coccidioides*, *Blastomyces*, and *Histoplasma* germinating conidia (36). Each of these dimorphic fungi represents a significant health threat for humans, and the dimorphism of these fungi is an important feature for their pathogenicity (37, 38). Activity was assessed based on 80% inhibition of growth (MIC_80 _per the CLSI M38-A2 standard); the positive control for these studies was voriconazole (Table 5). MM-TM, DM-TM, and NM have MIC_80_ values of <4 µg/ml for all strains of Histoplasma capsulatum, Blastomyces dermatitidis, and *Coccidioides* spp. assayed.

**TABLE 5:** MIC results for MM-TM, DM-TM, and NM against dimorphic fungi

Species	Isolate	MIC_80_ (µg/ml)^a^			
		NM	MM-TM	DM-TM	VOR^b^
*Coccidioides* sp.	Cocci1	1	2	2	0.25
	Cocci2	1	2	2	0.125
	Cocci3	0.5	1	1	0.06
	Cocci5	2	2	1	0.125
	Cocci6	1	1	1	0.25
	Cocci7	1	1	1	0.125
	Cocci8	1	1	1	0.125
	Cocci9	1	2	2	1
	Cocci10	1	1	1	0.06

Blastomyces dermatitidis	BD1	1	2	1	≤0.03
	BD2	1	2	1	≤0.03
	BD3	1	2	1	0.5

Histoplasma capsulatum	HC1	4	2	1	≤0.03
	HC2	1	1	0.5	0.25
	HC3	4	2	2	0.125
	HC4	2	2	1	0.06
	HC5	2	2	1	0.25
	HC6	4	2	1	0.125
	HC7	2	2	1	0.5
	HC8	2	1	1	0.25
	HC9	4	2	2	0.06
	HC10	4	2	2	0.06

a MIC_80_, MIC required to halt 80% of growth.

b VOR, voriconazole.

### NM, MM-TM, and DM-TM have moderate antifungal activity against *Pneumocystis* spp. 

*Pneumocystis* spp. are known to cause lethal pneumonia in immunocompromised patients and are particularly associated with AIDS patients. Relatively little is known about *Pneumocystis* spp., ranging from its life cycle to survival strategies within mammalian hosts, since there currently exists no way to culture these species in a laboratory setting. Antifungal activity of the three polymers was assessed against cryopreserved and characterized *Pneumocystis* spp. Fungal viability in the presence of each polymer was measured using an ATP production assay (ATP-liteM assay) after 24, 48, and 72 h of polymer exposure. Calculating percent ATP reduction for all samples allowed us to determine 50% inhibitory concentrations (IC_50_s) (Table 6 and Tables S3 and S4). MM-TM and DM-TM exhibited moderate activity, with 72 h IC_50_ values ranging from 2 to 5 µg/ml. The homopolymer NM was less active against Pneumocystis murina compared to Pneumocystis carinii, with 72-h IC_50_ values of 15 and 3 µg/ml, respectively. These data illustrate that even noncanonical fungi with unusual adaptations and niches (*Pneumocystis* species were once thought to be protozoan parasites [39]) show sensitivity to nylon-3 polymers, expanding opportunities for the use of nylon-3 materials against diverse fungi.

10.1128/mSphere.00223-18.5 TABLE S3  Pneumocystis carinii ATP-liteM results. Download TABLE S3, PDF file, 0.1 MB. Copyright © 2018 Rank et al. 2018 Rank et al. This content is distributed under the terms of the Creative Commons Attribution 4.0 International license.

10.1128/mSphere.00223-18.6 TABLE S4  Pneumocystis murina ATP-liteM results. Download TABLE S4, PDF file, 0.1 MB. Copyright © 2018 Rank et al. 2018 Rank et al. This content is distributed under the terms of the Creative Commons Attribution 4.0 International license.

**TABLE 6:** IC_50_ results for MM-TM, DM-TM, and NM against *Pneumocystis* spp.

Species	IC_50_ (µg/ml)^a^			Activity scale of day 3 IC_50_ values (µg/ml)
	NM	MM-TM	DM-TM	
Pneumocystis carinii	3.4	4.5	4.8	Moderate (1.0–9.99)
Pneumocystis murina	15	3.8	2.3	Slight (10.0–49.9)

a IC_50_, 50% inhibitory concentration.

## DISCUSSION

**FIG 2:**
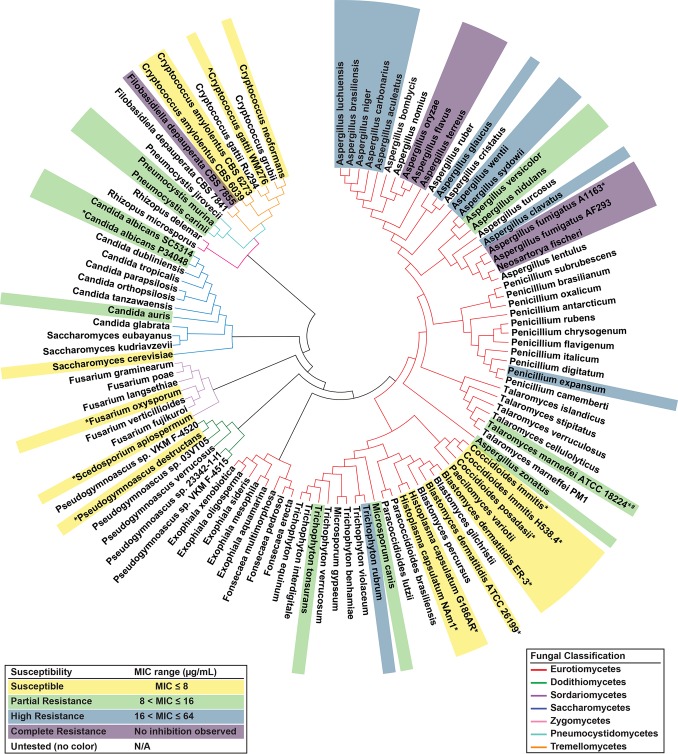
Maximum likelihood phylogeny of fungal species used in this study. Species were color coded based on their general sensitivity to MM-TM, DM-TM, and NM nylon-3 polymers. All three polymers showed roughly equivalent activity against highly diverse fungi across the fungal kingdom. Any strain for which polymer activity was not equivalent across all three polymers is indicated by the pound symbol. Any species assessed in this study in which a different strain was used to compose the phylogeny tree is indicated by an asterisk. The strain tested in a previous publication (11) is indicated by a caret. N/A, not available.

Our data show that the nylon-3 polymers MM-TM, DM-TM, and NM are effective against a surprisingly broad spectrum of fungi (Fig. 2), with only low to moderate toxicity toward mammalian cells (see Text S1 and Tables S5 and S6 in supplemental material). Here we were able to assess sensitivity of 18 pathogenic genera toward the nylon-3 chemotype, based on measurements with 41 species and 72 isolates. Visualization of our results in the context of fungal phylogeny indicates general sensitivity to the polymers, with notable exceptions in the *Aspergillus* clade and the basidiomycete Filobasidiella depauperata (Fig. 2). Overall, these data provide support for the idea that nylon-3 polymers could be useful as broad-action therapeutic agents.

10.1128/mSphere.00223-18.1 TEXT S1  Mammalian cell toxicity assays. Download TEXT S1, PDF file, 0.2 MB. Copyright © 2018 Rank et al. 2018 Rank et al. This content is distributed under the terms of the Creative Commons Attribution 4.0 International license.

10.1128/mSphere.00223-18.7 TABLE S5  Polymer toxicity on mammalian cells: A549 ATP-liteM results. Download TABLE S5, PDF file, 0.1 MB. Copyright © 2018 Rank et al. 2018 Rank et al. This content is distributed under the terms of the Creative Commons Attribution 4.0 International license.

10.1128/mSphere.00223-18.8 TABLE S6  Polymer toxicity on mammalian cells: L2 ATP-liteM results. Download TABLE S6, PDF file, 0.1 MB. Copyright © 2018 Rank et al. 2018 Rank et al. This content is distributed under the terms of the Creative Commons Attribution 4.0 International license.

Activity of the nylon-3 polymers was observed against several species of fungi with limited or ineffective treatment options. For instance, the nylon-3 polymers were active against Rhizopus arrhizus, one of the causative agents of mucormycosis, which is a life-threatening disease in both immunocompetent and immunocompromised patients (40). Depending on the predisease status of the patient and route of infection, mucormycosis may present in pulmonary, rhino-orbital-cerebral, cutaneous, gastrointestinal, or disseminated forms. Treatment of mucormycosis often requires administration of amphotericin B after surgical debridement of necrotic tissues (40). Even with rigorous treatment regimes, mortality rates are high (>40%), and amphotericin B toxicity is problematic for patients. The sensitivity of R. arrhizus to nylon-3 polymers suggests a new strategy to combat a challenging and deadly fungal disease for which current treatment options are highly limited (40).

All three nylon-3 polymers also showed efficacy against dermatophytes as preestablished hyphae. It is estimated that 20 to 25% of the world’s population has skin mycoses (41), and while these infections are often superficial and cosmetic in nature, they can have severe impact on quality of life. Topical treatment of dermatophyte infections relies mostly on two classes of antifungal drugs, the azoles (e.g., fluconazole and ketoconazole) and the allylamines (e.g., terbinafine and naftifine). However, there have been increasing incidences of resistant and refractory dermatophyte infections in the clinic, and daily dosages of terbinafine can reach 1,000 mg (42, 43). Nylon-3 polymers, with their ease of synthesis and low anticipated cost of production, could provide a viable option for treating such topical fungal infections.

Comparisons between sensitive and resistant genera may ultimately enable us to identify the origin(s) of sensitivity and to elucidate mechanisms of nylon-3 action. A particular opportunity to understand how nylon-3 polymers confer antifungal activity emerges from the observation that these materials were ineffective against Filobasidiella depauperata, despite the polymers’ strong activity against closely related *Cryptococcus* species. Since the polymers were active against other filamentous fungi, it is likely that properties not associated with filamentous morphology are responsible for this polymer resistance phenotype. Genetic comparisons of C. neoformans and F. depauperata may reveal insights into the mechanisms by which nylon-3 polymers exert their antifungal effects.

While all three nylon-3 polymers studied, NM, MM-TM, and DM-TM, had similar antifungal activities, subtle differences in activity profiles were noted throughout our phylogenetically broad analysis. NM was the most active polymer against the ascomycete yeasts *Candida* and *Saccharomyces*, while DM-TM was the most potent polymer against filamentous fungi. Overall hydrophobicity represents one of the main chemical properties that varied among the polymers (NM < MM-TM < DM-TM). All subunits in NM bear a short cationic side chain. Therefore, this homopolymer therefore cannot display a large and well-defined hydrophobic surface, which is believed to be an important feature of host defense peptides (HDPs) and their synthetic mimics. The MM-TM and DM-TM copolymers, on the other hand, should manifest greater overall hydrophobicity relative to NM because there are one or two additional nonpolar CH_2_ units in the MM and DM cationic subunits relative to NM, and each of the copolymers contains 20 to 30% of the entirely hydrophobic TM subunit. The copolymer expected to be most hydrophobic, DM-TM, was the most active among the three against filamentous fungi; however, this enhanced activity comes at the cost of increased mammalian cell toxicity relative to MM-TM or NM (Text S1 and Tables S5 and S6). The subtle differences in antifungal activity across the three polymers suggest that increased overall hydrophobicity may be required to enhance activity against filamentous fungi, particularly of the *Aspergillus* clade. Polymers with increased hydrophobicity relative to those discussed here, however, may require additional design features to avoid enhanced toxicity toward eukaryotic cells. Here we present the activities of materials with a novel chemotype, nylon-3 polymers, against a varied array of fungi from across the fungal kingdom. The surprisingly broad spectrum of nylon-3 antifungal activities, including inhibition of difficult-to-treat human pathogens, offers promise for the development of polymeric compounds with therapeutically useful properties. The ease of synthesis and structural diversification of nylon-3 polymers provide a broad scope for future efforts to optimize activity against pathogenic fungi while limiting toxicity toward the host.

## MATERIALS AND METHODS

### Polymer synthesis and characterization.

All polymers and monomers were prepared using previously reported methods (20–23). Please see the supplemental material for more information about polymer synthesis and characterization methods (Text S2 and Table S7).

10.1128/mSphere.00223-18.2 TEXT S2  Polymer synthesis and characterization. Download TEXT S2, PDF file, 0.1 MB. Copyright © 2018 Rank et al. 2018 Rank et al. This content is distributed under the terms of the Creative Commons Attribution 4.0 International license.

10.1128/mSphere.00223-18.9 TABLE S7  Polymer batch characterization. Download TABLE S7, PDF file, 0.1 MB. Copyright © 2018 Rank et al. 2018 Rank et al. This content is distributed under the terms of the Creative Commons Attribution 4.0 International license.

### CLSI M27-A3 protocol.

The MIC_100_ endpoint of each antifungal agent was determined as the lowest concentration to inhibit 100% of fungal growth compared to the no-drug control. MIC_100_ values for all yeasts assayed (Table 7) were determined by the broth microdilution method according to the CLSI M27-A3 guidelines, with slight modifications (25). Briefly, fungal cells at a density of 0.5 × 10^3^ to 2.5 × 10^3^ cells/ml were incubated at 30 to 35°C in RPMI 1640 plus 0.145 M 3-(*N*-morpholino)propanesulfonic acid (MOPS) (pH 7.0) in 96-well plates with twofold serial dilutions of nylon-3 polymer or fluconazole (FLC) from 1 to 64 µg/ml. After 24 to 72 h, the optical density at 600 nm (OD_600_) of each well was measured using a microplate reader. Wells containing fungal cells with no drug and wells containing only RPMI 1640 were used as positive and blank controls, respectively. Percent cell growth was determined as [(sample absorbance − blank absorbance)/(control absorbance − blank absorbance)] × 100%. All values reported represent the average MIC_100_ value for more than two biological replicates and two or more technical replicates each. The average MIC_100_ value consistently fell within a twofold serial dilution of the concentration of each experimental replicate. Any modifications to this protocol for a specific species is listed in Table 7.

**TABLE 7:** Strains used in this study

Species	Isolate or strain	Source	Description	Antifungal test^a^
S. cerevisiae	W303 (ATCC 200060)	Lab strain, obtained from Catherine Fox lab	*leu2-3*,*112 trp1-1 can1-100 ura3-1 ade2-1 his3-11*,*15*	CLSI M27-A3, 1 mM uracil
C. neoformans	CN1	Cerebrospinal fluid; clinical isolate	Azole susceptible	CLSI M27-A3, tested by NIAID
	CN2	Popliteal lymph node; clinical isolate (animal)	Fluconazole resistant	CLSI M27-A3, tested by NIAID
	CN3	Cerebrospinal fluid; clinical isolate	Fluconazole resistant	CLSI M27-A3, tested by NIAID
C. amylolentus	CBS 6273	Insect frass isolate, obtained from Joseph Heitman lab		CLSI M27-A3
	CBS 6039	Insect frass isolate, obtained from Joseph Heitman lab		CLSI M27-A3
C. albicans	ATCC 90028 (CA1)	Blood		CLSI M27-A3, tested by NIAID
	CA2			CLSI M27-A3, tested by NIAID
	CA3	Blood	Azole resistant	CLSI M27-A3, tested by NIAID
C. krusei	QC		CLSI QC isolate for susceptibility testing	CLSI M27-A3, tested by NIAID
C. auris	B11220	Clinical isolate (Japan), obtained from David Andes lab		CLSI M27-A3
	C54039	Clinical isolate (Columbia), obtained from David Andes lab	Amphotericin B resistant	CLSI M27-A3
*Coccidioides* sp.	Cocci1	Unknown; NIAID preclinical testing services		CLSI M38-A2, tested by NIAID
	Cocci2	Unknown; NIAID preclinical testing services		CLSI M38-A2, tested by NIAID
	Cocci3	Unknown; NIAID preclinical testing services		CLSI M38-A2, tested by NIAID
	Cocci4	Unknown; NIAID preclinical testing services		CLSI M38-A2, tested by NIAID
	Cocci5	Unknown; NIAID preclinical testing services		CLSI M38-A2, tested by NIAID
	Cocci6	Unknown; NIAID preclinical testing services		CLSI M38-A2, tested by NIAID
	Cocci7	Unknown; NIAID preclinical testing services		CLSI M38-A2, tested by NIAID
	Cocci8	Unknown; NIAID preclinical testing services		CLSI M38-A2, tested by NIAID
	Cocci9	Unknown; NIAID preclinical testing services		CLSI M38-A2, tested by NIAID
	Cocci10	Unknown; NIAID preclinical testing services		CLSI M38-A2, tested by NIAID
B. dermatitidis	BD1	Unknown; NIAID preclinical testing services		CLSI M38-A2, tested by NIAID
	BD2	Unknown; NIAID preclinical testing services		CLSI M38-A2, tested by NIAID
	BD3	Unknown; NIAID preclinical testing services		CLSI M38-A2, tested by NIAID
H. capsulatum	HC1	Unknown; NIAID preclinical testing services		CLSI M38-A2, tested by NIAID
	HC2	Unknown; NIAID preclinical testing services		CLSI M38-A2, tested by NIAID
	HC3	Unknown; NIAID preclinical testing services		CLSI M38-A2, tested by NIAID
	HC4	Unknown; NIAID preclinical testing services		CLSI M38-A2, tested by NIAID
	HC5	Unknown; NIAID preclinical testing services		CLSI M38-A2, tested by NIAID
	HC6	Unknown; NIAID preclinical testing services		CLSI M38-A2, tested by NIAID
	HC7	Unknown; NIAID preclinical testing services		CLSI M38-A2, tested by NIAID
	HC8	Unknown; NIAID preclinical testing services		CLSI M38-A2, tested by NIAID
	HC9	Unknown; NIAID preclinical testing services		CLSI M38-A2, tested by NIAID
	HC10	Unknown; NIAID preclinical testing services		CLSI M38-A2, tested by NIAID
A. fumigatus	CEA10	Nancy Keller lab		CLSI M38-A2, 35°C
A. flavus	NRRL3357	Nancy Keller lab		CLSI M38-A2, 35°C
A. oryzae	Rib40	JGI Genome Project		CLSI M38-A2, 35°C
A. terreus	NCCB IH2624	JGI Genome Project		CLSI M38-A2, 35°C
A. parasiticus	Su-1	JGI Genome Project		CLSI M38-A2, 35°C
N. fisheri	CBS 544.65	JGI Genome Project		CLSI M38-A2, 35°C
A. nidulans	FGSCA4	JGI Genome Project		CLSI M38-A2, 35°C
A. aculeatus	CBS 172.66	JGI Genome Project		CLSI M38-A2, 35°C
A. carbonarius	DT0115-B6	JGI Genome Project		CLSI M38-A2, 35°C
A. wentii	DT0136-E9	JGI Genome Project		CLSI M38-A2, 35°C
A. sydowii	CBS 593.65	JGI Genome Project		CLSI M38-A2, 35°C
A. foetidus	CBS 106.47	JGI Genome Project		CLSI M38-A2, 35°C
A. zonatus	CBS 506.65	JGI Genome Project		CLSI M38-A2, 35°C
A. niger	CBS 113.46	JGI Genome Project		CLSI M38-A2, 35°C
A. glaucus	CBS 516.65	JGI Genome Project		CLSI M38-A2, 35°C
A. brasiliensis	CBS 101740	JGI Genome Project		CLSI M38-A2, 35°C
A. clavatus	CBS 513.65	JGI Genome Project		CLSI M38-A2, 35°C
A. versicolor	CBS 795.97	JGI Genome Project		CLSI M38-A2, 35°C
P. expansum	d1	Apples from Israel in 2012		CLSI M38-A2, 29°C
T. marneffei	FRR2161, CBS 334.59, ATCC 18224	ATCC		CLSI M38-A2, 29°C
P. variotii	QC		CLSI QC isolate	CLSI M38-A2, tested by NIAID
F. oxysporum	FO1	Blood	Clinical isolate	CLSI M38-A2, tested by NIAID
	FO_2_	Bone	Clinical isolate	CLSI M38-A2, tested by NIAID
	FO3	Blood	Clinical isolate	CLSI M38-A2, tested by NIAID
S. apiospermum	SA1	Toe	Clinical isolate	CLSI M38-A2, tested by NIAID
	SA2	Elbow tissue	Clinical isolate	CLSI M38-A2, tested by NIAID
L. prolificans	LP1	Chest wound	Clinical isolate	CLSI M38-A2, tested by NIAID
R. arrhizus	RA1	Nose tissue	Clinical isolate	CLSI M38-A2, tested by NIAID
	RA2	Tissue upper extremity	Clinical isolate	CLSI M38-A2, tested by NIAID
	RA3	Palate tissue	Clinical isolate	CLSI M38-A2, tested by NIAID
F. depauperata	CBS 7855	Caterpillar isolate, obtained from Joseph Heitman lab		CLSI M38-A2, SD was used as the growth medium during antifungal testing
T. rubrum	ATCC 28188	Alana Sterkel lab; UW^b^ Department of Pathology and Laboratory Medicine	Clinical isolate; nail	CLSI M38-A with modifications^c^
M. canis	UW10	Karen Moriello lab; UW School of Veterinary Medicine	Wild-animal isolate; cat	CLSI M38-A with modifications^c^
T. tonsurans	CBS 112818	Theodore White lab, Broad Institute	Clinical isolate; cheek	CLSI M38-A with modifications^c^
P. destructans	ATCC MYA-4855	Jeffrey Lorch lab; U.S. Geological Survey	Wild-animal isolate; bat	CLSI M38-A with modifications^c^

a The antifungal test and modifications are given. NIAID, National Institute of Allergy and Infectious Diseases.

b UW, University of Wisconsin.

c CLSI M38-A with modifications to test established hyphae rather than conidia for susceptibility. See the paragraph on the revised CLSI M38-A protocol for hyphae of filamentous fungi and dermatophytes in Materials and Methods.

### Revised CLSI M38-A protocol for hyphae of filamentous fungi and dermatophytes.

The MIC_100_ endpoint of each antifungal agent was determined as the lowest concentration to inhibit 100% of hyphal growth. MIC_100_ values for all filamentous fungi assayed were determined by the broth microdilution method according to CLSI M38-A guidelines, with modifications (44).

For the dermatophytes, crushed hyphal fragments of Trichophyton tonsurans, Trichophyton rubrum, and Microsporum canis at a density corresponding to an OD_600_ reading of 0.05 were incubated at 29°C in RPMI 1640 plus 0.145 M MOPS, pH 7.0, in 96-well plates with twofold serial dilutions of nylon-3 polymer or with itraconazole (ITRA) from 1 to 64 µg/ml. After 96 h, the MIC of each well was measured by monitoring the changes in OD_600 _compared to the value at the 0-h time point. From the OD measurements, a difference in OD greater than 0.03 from the values at the 96- and 0-h time points was indicative of growth. All values reported represent the average MIC_100 _value for two biological replicates and six technical replicates each. The average MICs consistently fell within a twofold serial dilution of the concentration of each experimental replicate.

Pseudogymnoascus destructans at a density of 1 × 10^4^ spores/ml was incubated at 12°C in RPMI 1640 plus 0.145 M MOPS, pH 7.0, in 96-well plates with twofold serial dilutions of nylon-3 polymer or with ITRA from 1 to 64 µg/ml. After 96 h, the MIC of each well was measured by monitoring changes in OD_600 _compared to the value at the 0-h time point. From the OD measurements, a difference in OD greater than 0.01 from the values at the 120- and 0-h time points was indicative of growth. All values reported represent the average MIC_100_ value for two biological replicates and six technical replicates each. The average MICs consistently fell within a twofold serial dilution of the concentration of each experimental replicate.

A total of 1 × 10^4^ conidia/ml of Aspergillus fumigatus, Aspergillus terreus, Aspergillus flavus, or Aspergillus nidulans were inoculated into RPMI 1640 plus 0.145 M MOPS, pH 7.0 in 96-well plates and incubated at 37°C for 18 to 24 h to allow for growth of the extensive hyphal network. Hyphae were then treated with twofold serial dilutions of nylon-3 polymer or with ITRA from 1 to 64 µg/ml and allowed to incubate at 37°C. OD_600_ readings were obtained at 0, 24, and 48 h. From the OD measurements, a difference in OD greater than 0.05 from the values at the 48- and 0-h time points was indicative of growth. All values reported represent the average MIC_100_ value for two biological replicates and six technical replicates each. The average MICs consistently fell within a twofold serial dilution of the concentration of each experimental replicate.

A total of 1 × 10^4^ spores/ml of Penicillium expansum conidia were inoculated into RPMI 1640 plus 0.145 M MOPS, pH 7.0, in 96-well plates and incubated at room temperature for 24 to 48 h to allow for germination into established hyphae. Hyphae were then treated with twofold serial dilutions of nylon-3 polymer or with ITRA from 1 to 64 µg/ml and allowed to incubate further at room temperature. OD_600_ readings were obtained at 0, 24, and 48 h. After 48 h, the MIC of each well was measured by monitoring changes in OD_600_ in comparison to the value at the 0-h time point. From the OD measurements, a difference in OD greater than 0.05 from the values at the 48- and 0-h time point was indicative of growth. All values reported represent the average MIC_100_ value for two biological replicates and six technical replicates each. The average MICs consistently fell within a twofold serial dilution of the concentration of each experimental replicate.

### CLSI M38-A2 protocol.

The MIC_100_ endpoint of each antifungal agent was determined as the lowest concentration to inhibit 100% of hyphal outgrowth from conidia. The MIC_80_ endpoint of each antifungal was determined as the concentration to inhibit 80% of fungal growth. MIC_100_ or MIC_80_ values for all filamentous or dimorphic fungi assayed (Table 7) were determined by the broth macrodilution method according to the CLSI M38-A2 guidelines, with slight modifications (36). Briefly, fungal cells at a density of 0.5 × 10^4 ^to 5 × 10^4^ conidia/ml were incubated at 35°C in RPMI 1640 plus 0.145 M MOPS, pH 7.0, in 96-well plates with twofold serial dilutions of the nylon-3 polymer or with fluconazole (FLC), voriconazole (VOR), or itraconazole (ITRA) from 1 to 64 µg/ml. After 24 to 72 h, the MIC of each well was measured visually using a dissecting microscope. All values reported represent the average MIC_100_ values for more than two biological replicates and two or more technical replicates each. The average MICs consistently fell within a twofold serial dilution of the concentration of each experimental replicate. Any modifications to this protocol for a specific species are listed in Table 7.

### ATP-liteM assay for *Pneumocystis* spp.

Cryopreserved and characterized Pneumocystis carinii isolated from rat lung tissue and Pneumocystis murina isolated from mouse lung tissue were distributed into triplicate wells of 48-well plates with a final volume of 500 µl at a final concentration of 5 × 10^7^ nuclei/ml P. carinii and 5 × 10^6^ nuclei/ml P. murina. Controls and compounds were added and incubated at 36°C and 5% CO_2_. At 24, 48, and 72 h, 10% of the well volume was removed, and the ATP content was measured using PerkinElmer ATP-liteM luciferin-luciferase assay. The luminescence generated by the ATP content of the samples was measured by a BMG PolarStar optima spectrophotometer. A sample of each group was examined microscopically on the final assay day to rule out the presence of bacteria.

For 50% inhibitory concentration (IC_50_) calculations, background luminescence was subtracted, and triplicate well readings of duplicate assays were averaged. For each day’s reading, percent reduction in ATP for all groups was calculated as follows: [(medium control − experimental value)/medium control] × 100. IC_50_ values were calculated using GraphPad Prism 6 linear regression program (Tables S3 and S4).

### Evolutionary analysis.

Multigene-based phylogeny between all fungal species depicted in Fig. 2 was constructed based on amino acid sequences of 14 genes (Table S8) identified in *Fusarium* spp. to be conserved for various energetic processes (45). Orthologous proteins for genes listed in Table S8 were identified in each species using HMMer (Ensembl Fungi). Ten genes from Wiemann et al. (45) were omitted from the analysis due to lack of orthologous protein identity in one or more of the species tested. Resulting protein sequences for each of the gene were aligned to well-conserved regions using MAFFT (46) and trimmed using Gblocks (47) using default parameters. All positions containing gaps and missing data were eliminated. Trimmed alignments were then concatenated for each species, and a maximum likelihood (ML) phylogeny was inferred based on the JTT model (48) using MEGA7 (49) and viewed in FigTree (http://tree.bio.ed.ac.uk/software/figtree/).

10.1128/mSphere.00223-18.10 TABLE S8  Fourteen genes used for evolutionary analysis of all fungal species depicted in Fig. 2 of the article. Download TABLE S8, PDF file, 0.1 MB. Copyright © 2018 Rank et al. 2018 Rank et al. This content is distributed under the terms of the Creative Commons Attribution 4.0 International license.

### Data availability.

All data are included in the article and supplemental files.
