# Supplementary material for: Peptide-Like Nylon-3 Polymers with Activity against Phylogenetically Diverse, Intrinsically Drug-Resistant Pathogenic Fungi
Source: mSphere. 2018 May 23;3(3):e00223-18. doi: 10.1128/mSphere.00223-18 (PMC5967195; doi:10.1128/mSphere.00223-18)
Supplement: TABLE S1 [file sph003182551st1.pdf]

Table S1

Antifungal agent	MIC$_{100}$ (µg/ml) *C. neoformans* JEC 20x21 yeast (1.25 x 10^3 cells/ml)	
	**RPMI, 48 h**	**SD, 48 h**
MM-TM	4	8
DM-TM	2	2
NM	2	4
Flu	2	4
AmB	0.125	0.25
