# Supplementary material for: Peptide-Like Nylon-3 Polymers with Activity against Phylogenetically Diverse, Intrinsically Drug-Resistant Pathogenic Fungi
Source: mSphere. 2018 May 23;3(3):e00223-18. doi: 10.1128/mSphere.00223-18 (PMC5967195; doi:10.1128/mSphere.00223-18)
Supplement: TABLE S2 [file sph003182551st2.pdf]

Table S2

Antifungal agent	**MIC$_{100}$ (µg/ml) *C. neoformans* JEC 20x21 spores (5 x 10^5 spores/ml)**	
	**RPMI, 15 h**	**SD, 15 h**
MM-TM	16	16
DM-TM	8	16
NM	4	16
Flu	>64	>64
AmB	<0.03	0.125
