# Supplementary material for: Peptide-Like Nylon-3 Polymers with Activity against Phylogenetically Diverse, Intrinsically Drug-Resistant Pathogenic Fungi
Source: mSphere. 2018 May 23;3(3):e00223-18. doi: 10.1128/mSphere.00223-18 (PMC5967195; doi:10.1128/mSphere.00223-18)
Supplement: TABLE S3 [file sph003182551st3.pdf]

Table S3

P. carinii % reduction in ATP/vehicle control[a]			
	24 h	48 h	72 h
Ampicillin 10 µg/ml	12.28	0	2.65
Pentamidine 1 µg/ml	86.79	87.62	95.23
NM			
100 µg/ml	54.44	77.13	99.13
10 µg/ml	34.01	28.65	55.91
1 µg/ml	11.91	14.73	28.21
0.1 µg/ml	15.54	14.18	19.79
IC_{50}			3.4 µg/ml
MM-TM			
100 µg/ml	77.36	88.89	89.99
10 µg/ml	49.58	35.99	43.43
1 µg/ml	30.18	17.47	29.86
0.1 µg/ml	18.01	10.80	23.75
IC_{50}			4.5 µg/ml
DM-TM			
100 µg/ml	95.95	95.55	99.54
10 µg/ml	10.78	36.81	42.82
1 µg/ml	2.64	0	35.30
0.1 µg/ml	0	0	0
IC_{50}			4.8 µg/ml

[a] Data provided by National Institute of Allergy and Infectious Diseases, NIAID
