# Supplementary material for: Peptide-Like Nylon-3 Polymers with Activity against Phylogenetically Diverse, Intrinsically Drug-Resistant Pathogenic Fungi
Source: mSphere. 2018 May 23;3(3):e00223-18. doi: 10.1128/mSphere.00223-18 (PMC5967195; doi:10.1128/mSphere.00223-18)
Supplement: TABLE S4 [file sph003182551st4.pdf]

Table S4

P. murina % reduction in ATP/vehicle control[a]			
	24 h	48 h	72 h
Ampicillin 10 µg/ml	0	0.9	0
Pentamidine 1 µg/ml	90.80	84.39	91.86
NM			
NM_{20} 100 µg/ml	90.37	93.30	91.82
10 µg/ml	48.35	18.36	25.15
1 µg/ml	26.85	10.90	0.41
0.1 µg/ml	31.61	6.85	0
IC_{50}			15 µg/ml
MM-TM			
100 µg/ml	94.54	93.05	90.65
10 µg/ml	56.48	57.99	79.30
1 µg/ml	28.30	25.4	17.08
0.1 µg/ml	14.54	25.18	2.01
IC_{50}			3.8 µg/ml
DM-TM			
100 µg/ml	94.57	96.00	97.09
10 µg/ml	44.98	49.31	92.66
1 µg/ml	23.32	20.95	15.31
0.1 µg/ml	16.76	17.38	13.21
IC_{50}			2.3 µg/ml

[a] Data provided by National Institute of Allergy and Infectious Diseases, NIAID
