# Supplementary material for: Peptide-Like Nylon-3 Polymers with Activity against Phylogenetically Diverse, Intrinsically Drug-Resistant Pathogenic Fungi
Source: mSphere. 2018 May 23;3(3):e00223-18. doi: 10.1128/mSphere.00223-18 (PMC5967195; doi:10.1128/mSphere.00223-18)
Supplement: TEXT S1 [file sph003182551s1.pdf]

Mammalian cell toxicity assays

Cultured adenocacincomic human alveolar basal epithelial cells, A549, and the murine T-cell hybridoma L2 cell line were plated at $2x10^5$ cells/ml and grown to confluent monolayers. Media was removed and replaced with fresh media containing controls and polymer dilutions. At 24, 48, and 72 h, media was aspirated from wells, adherent cells were lysed with 0.1 M NaOH, and a portion of the lysate was measured for ATP content using Perkin Elmer ATP-liteM luciferin-luciferase assay. The luminescence generated by the ATP content of the samples was measured by a BMG PolarStar optima spectrophotometer. For 50 percent inhibitory concentrations (IC_{50}) calculations, background luminescence was subtracted and triplicate well readings of triplicate assays were averaged. For each day's reading, percent reduction in ATP for all groups was calculated as follows: (media control - experimental)/ media control x100. 50% inhibitory concentration (IC_{50}) was calculated using INSTAT/PRIZM linear regression program.

MM-TM, DM-TM and NM were found to be non-toxic (IC_{50} > 100 μg/ml) against the A549 cell line. More variability in toxicity was observed against the L2 cell line, in which trends followed those of previously published nylon-3 toxicity data (1, 3, 4). Against the L2 cell line, the cationic homopolymer, NM, was nontoxic (IC_{50} > 100 μg/ml), while the more hydrophobic MM-TM and DM-TM copolymers exhibited mild to moderate toxicity against this cell line (IC_{50} = 22 and 7 μg/ml, respectively).
