# Supplementary material for: Peptide-Like Nylon-3 Polymers with Activity against Phylogenetically Diverse, Intrinsically Drug-Resistant Pathogenic Fungi
Source: mSphere. 2018 May 23;3(3):e00223-18. doi: 10.1128/mSphere.00223-18 (PMC5967195; doi:10.1128/mSphere.00223-18)
Supplement: TABLE S5 [file sph003182551st5.pdf]

Table S5

A549 % reduction in ATP/vehicle control[a]			
	24 h	48 h	72 h
Antiymycin A 75 µg/ml	8.43 +/- 3.55	47.36 +/- 8.40	96.59 +/- 1.35
DM-TM – not toxic			
100 µg/ml	9.88 +/- 7.28	25.73 +/- 5.18	33.01 +/- 5.43
10 µg/ml	5.95 +/- 4.76	0	3.64 +/- 5.64
1 µg/ml	5.55 +/- 2.64	0	2.63 +/- 2.79
0.1 µg/ml	0	0	1.60 +/- 2.47
IC_{50}[b]			> 100 µg/ml
NM – not toxic			
100 µg/ml	15.84 +/-5.24	26.01 +/- 10.51	33.06 +/- 5.65
10 µg/ml	6.99 +/- 10.82	0	5.70 +/- 5.75
1 µg/ml	0	0	1.91 +/- 2.96
0.1 µg/ml	0	0	0.79 +/- 1.22
IC_{50}[b]			> 100 µg/ml
MM-TM – not toxic			
100 µg/ml	16.41 +/- 4.90	38.72 +/- 5.61	48.69 +/- 4.31
10 µg/ml	0	0	6.02 +/-9.30
1 µg/ml	0	0	5.40 +/- 8.06
0.1 µg/ml	0	0	0.32 +/- 0.49
IC_{50}[b]			> 100 µg/ml

[a] Data provided by National Institute of Allergy and Infectious Diseases, NIAID. [b] Fifty percent inhibitory concentration (IC_{50})
