# Supplementary material for: Peptide-Like Nylon-3 Polymers with Activity against Phylogenetically Diverse, Intrinsically Drug-Resistant Pathogenic Fungi
Source: mSphere. 2018 May 23;3(3):e00223-18. doi: 10.1128/mSphere.00223-18 (PMC5967195; doi:10.1128/mSphere.00223-18)
Supplement: TABLE S6 [file sph003182551st6.pdf]

Table S6

L2 % reduction in ATP/vehicle control[a]			
	24 h	48 h	72 h
Antiymycin A 75 µg/ml	53.14 +/- 7.30	76.82 +/- 1.42	85.67 +/- 1.05
DM-TM – moderate toxicity			
100 µg/ml	97.54 +/- 0.07	99.55 +/- 0.02	99.67 +/- 0.04
10 µg/ml	3.20 +/- 4.96	5.27 +/- 4.09	39.47 +/- 4.06
1 µg/ml	0	2.48 +/- 3.85	13.71 +/- 19.64
0.1 µg/ml	0	0.25 +/- 0.38	0
IC_{50}[b]			7.29 µg/ml
NM– not toxic			
100 µg/ml	23.46 +/-10.23	59.90 +/- 5.38	64.80 +/- 1.53
10 µg/ml	5.02 +/- 7.03	0	7.10 +/- 5.48
1 µg/ml	4.72 +/- 7.32	0	8.85
0.1 µg/ml	0.51 +/- 0.80	0	0
IC_{50}[b]			> 100 µg/ml
MM-TM – mild toxicity			
100 µg/ml	82.67 +/- 1.35	98.38 +/- 0.21	99.15 +/- 0.31
10 µg/ml	3.71 +/- 5.75	0.21 +/- 0.32	0.75 +/-8.27
1 µg/ml	0.96 +/- 1.48	0	0
0.1 µg/ml	1.67 +/- 2.58	0.53 +/- 0.82	0
IC_{50}[b]			21.82 µg/ml

[a] Data provided by National Institute of Allergy and Infectious Diseases, NIAID. [b] Fifty percent inhibitory concentration (IC_{50})
