# Supplementary material for: Peptide-Like Nylon-3 Polymers with Activity against Phylogenetically Diverse, Intrinsically Drug-Resistant Pathogenic Fungi
Source: mSphere. 2018 May 23;3(3):e00223-18. doi: 10.1128/mSphere.00223-18 (PMC5967195; doi:10.1128/mSphere.00223-18)
Supplement: TEXT S2 [file sph003182551s2.pdf]

## Polymer synthesis and characterization

All polymers and monomers were prepared using previously reported methods (1–4). Polymers at the protected stage (with Boc protection of the side chain amine groups) were subjected to gel permeation chromatography characterization using either N,N-dimethylacetamide (DMAc) or tetrahydrofuran (THF) as the mobile phase. Side-chain deprotected polymers were characterized by Proton nuclear magnetic resonance (1H NMR) spectroscopy.

DMAc GPC analysis involved two Waters styragel HR 4E columns (particle size 5 μm) linked in series on a Waters PC instrument equipped with a refractive index detector (Waters 2410). DMAc containing 10 mM LiBr was used as the mobile phase at a flow rate of 1 mL/min at 80˚C. Number-average molecular weight (Mn) and dispersity (Đ) were calculated using Empower software and calibration curves from polymethacrylate standards in DMAc (10 mM LiBr at 80˚C). Polymer solutions (2.5 μg/ml in DMAc) were filtered through 0.2 μm polytetrafluroethylene filter before GPC analysis.

THF GPC analysis involved two Waters columns (Styragel HR 4E and HR 2 particle size 5 μm) linked in series. The Waters liquid chromatography unit (Alliance) was equipped with a multiangle light scattering detector (Wyatt miniDAWN TREOS, 658 nm) and a refractive index detector (Wyatt Optilab-rEX, 658 nm). Mn and Đ were calculated using ASTRA software using a dn/dc value of 0.1 mL/g.

The degree of polymerization ($Dp_{GPC}$), or average polymer chain length, was calculated based on the determined Mn value, NMR-determined average ratio of subunits per polymer chain, and the molecular weights of the subunits using the equation below,

$$Dp_{GPC} = (M_n - M_{eg})/[M_{cationic}x + M_{hydrophobic}(1 - x)]$$

where $M_{eg}$ is the mass of the N-terminal end group (*t*-$BuC_6H_4$-CO-), $M_{cationic}$ is the mass of the side-chain-protected cationic subunit, $M_{hydrophobic}$ is the mass of the hydrophobic subunit, and *x* is the mole fraction of the cationic subunit as determined via NMR analysis.

Each deprotected polymer was dissolved in $D_2O$ at a concentration of 8 mg/mL. Proton nuclear magnetic resonance ($^1H$ NMR) spectra were collected on a Bruker Avance III spectrometer at 400 MHz or 500 MHz at ambient temperature. Polymer spectra are reported in ppm using the resonance for residual protio solvent as the internal standard (4.790 ppm). The degree of polymerization ($Dp_{NMR}$) for each polymer was calculated from NMR integration of proton resonances. The aromatic resonances between 7.4 and 7.7 ppm of the N-terminal p-*t*-butylbenzoyl moiety were used for normalization to one polymer chain with an integration of 4H.

See examples below the NMR spectra provided for each polymer for sample $Dp_{NMR}$ and subunit composition calculations.

## References


1 Liu R, Chen X, Hayouka Z, Chakraborty S, Falk SP, Weisblum B, Masters KS, Gellman SH. 2013. Nylon-3 Polymers with Selective Antifungal Activity. J Am Chem Soc 135:5270–5273.

2 Liu R, Chen X, Falk SP, Mowery BP, Karlsson AJ, Weisblum B, Palecek SP, Masters KS, Gellman SH. 2014. Structure–Activity Relationships among Antifungal Nylon-3 Polymers: Identification of Materials Active against Drug-Resistant Strains of Candida albicans. J Am Chem Soc 136:4333–4342.

3 Rank LA, Walsh NM, Liu R, Lim FY, Bok JW, Huang M, Keller NP, Gellman SH, Hull CM. 2017. A cationic polymer that shows high antifungal activity against diverse human pathogens. Antimicrob Agents Chemother AAC.00204-17.

4 Liu R, Chen X, Chakraborty S, Lemke JJ, Hayouka Z, Chow C, Welch RA, Weisblum B, Masters KS, Gellman SH. 2014. Tuning the Biological Activity Profile of Antibacterial Polymers via Subunit Substitution Pattern. J Am Chem Soc 136:4410–4418.
