# Supplementary material for: Peptide-Like Nylon-3 Polymers with Activity against Phylogenetically Diverse, Intrinsically Drug-Resistant Pathogenic Fungi
Source: mSphere. 2018 May 23;3(3):e00223-18. doi: 10.1128/mSphere.00223-18 (PMC5967195; doi:10.1128/mSphere.00223-18)
Supplement: TABLE S7 [file sph003182551st7.pdf]

Table S7

Polymer	Batch	GPC characterization[a,b]			NMR characterization		
		$Đ_{GPC}$ [c]	Mn_{GPC} [d]	Dp_{GPC} [e]	Dp_{NMR} [f]	Observed subunit ratio (**MM:TM**)[g]	Mn_{NMR} [h]
MM-TM	1[a]	1.16	4728	24	16	75 : 25	3241
	2[b]	1.07	4833	24	14	79 : 21	2905
	3[a]	1.19	4202	20	13	85 : 15	2777
	4[a]	1.23	3898	19	16	75 : 25	3241
DM-TM	A[a]	1.13	5454	27	20	70 : 30	4120
	B[a]	1.15	4703	24	17	65 : 35	3440
	C[a]	1.11	4042	21	21	57 : 43	4042
	D[b]	1.05	4713	24	14	64 : 36	2848
NM	α[a]	1.29	2102	10	23	100	5086
	β[a]	1.21	2349	11	23	100	5086

[a]Side-chain-protected polymer characterization by gel permeation chromatography (GPC) using *N,N*-dimethylacetamide (DMAc) as the mobile phase. [b]Side-chain-protected polymer characterization by GPC using tetrahydrofuran (THF) as the mobile phase. [c]Dispersity. [d]The number average molecular weight of side-chain-protected polymers. [e]The degree of polymerization, or average polymer chain length, as calculated from $Mn_{GPC.}$ using the subunit ratios determined from Nuclear Magnetic Resonance (NMR) analysis. [f]The degree of polymerization, or average polymer chain length, as calculated by NMR integrations based on end group analysis, setting the *tert*-butyl benzoyl aromatic protons to an integration of one polymer chain. [g]Monomer subunit ratio of a polymer was calculated from NMR integrations, setting the *tert*-butyl benzoyl aromatic protons to an integration representative of one polymer chain. [h]The number average molecular weight of side-chain deprotected polymers, using NMR calculated subunit ratios.
