# Supplementary material for: Peptide-Like Nylon-3 Polymers with Activity against Phylogenetically Diverse, Intrinsically Drug-Resistant Pathogenic Fungi
Source: mSphere. 2018 May 23;3(3):e00223-18. doi: 10.1128/mSphere.00223-18 (PMC5967195; doi:10.1128/mSphere.00223-18)
Supplement: TABLE S8 [file sph003182551st8.pdf]

Table S8

Code	Protein ID	Predicted Function
P1	FFUJ_01475	glucose-6-phosphate dehydrogenase
P2	FFUJ_01340	vacuolar ATP synthase subunit H
P3	FFUJ_05032	Kinesin
P8	FFUJ_04170	pyruvate dehydrogenase (lipoamide) alpha chain precursor
P9	FFUJ_02452	myosin
P12	FFUJ_02998	ATP3-F1F0-ATPase complex
P19	FFUJ_08584	pyruvate dehydrogenase
P20	FFUJ_08541	Mitochondrial oxaloacetate/sulfate carrier protein
P21	FFUJ_08319	Cytochrome c
P22	FFUJ_08315	beta-succinyl CoA synthetase precursor protein
P25	FFUJ_13760	ATP-specific succinyl-coa synthetase alpha subunit
P26	FFUJ_09852	mitochondrial serine--tRNA ligase
P27	FFUJ_09776	H+-transporting ATP synthase beta chain
P28	FFUJ_09575	NDE1-mitochondrial cytosolically directed NADH dehydrogenase
